# Navigating the Murine Brain: Toward Best Practices for Determining and Documenting Neuroanatomical Locations in Experimental Studies

**DOI:** 10.3389/fnana.2018.00082

**Published:** 2018-11-02

**Authors:** Ingvild E. Bjerke, Martin Øvsthus, Krister A. Andersson, Camilla H. Blixhavn, Heidi Kleven, Sharon C. Yates, Maja A. Puchades, Jan G. Bjaalie, Trygve B. Leergaard

**Affiliations:** Department of Molecular Medicine, Institute of Basic Medical Sciences, University of Oslo, Oslo, Norway

**Keywords:** best practice, brain atlas, data mining, data sharing, FAIR, reproducibility, location metadata, rodent brain

## Abstract

In experimental neuroscientific research, anatomical location is a key attribute of experimental observations and critical for interpretation of results, replication of findings, and comparison of data across studies. With steadily rising numbers of publications reporting basic experimental results, there is an increasing need for integration and synthesis of data. Since comparison of data relies on consistently defined anatomical locations, it is a major concern that practices and precision in the reporting of location of observations from different types of experimental studies seem to vary considerably. To elucidate and possibly meet this challenge, we have evaluated and compared current practices for interpreting and documenting the anatomical location of measurements acquired from murine brains with different experimental methods. Our observations show substantial differences in approach, interpretation and reproducibility of anatomical locations among reports of different categories of experimental research, and strongly indicate that ambiguous reports of anatomical location can be attributed to missing descriptions. Based on these findings, we suggest a set of minimum requirements for documentation of anatomical location in experimental murine brain research. We furthermore demonstrate how these requirements have been applied in the EU Human Brain Project to optimize workflows for integration of heterogeneous data in common reference atlases. We propose broad adoption of some straightforward steps for improving the precision of location metadata and thereby facilitating interpretation, reuse and integration of data.

## Introduction

Over last decades, considerable effort has been invested in large-scale the production of neuroscience data (see, e.g., Stopps et al., 2004; Boy et al., 2006; Lein et al., 2007; Zakiewicz et al., 2011; Hintiryan et al., 2012; Ragan et al., 2012; Oh et al., 2014). With increasingly efficient data production pipelines the number of scientific reports and amount of available data is steadily growing (Hey and Trefethen, 2003). To organize, compare and integrate such large amounts of data into new knowledge and understanding about the brain, new computational approaches have emerged (Amari et al., 2002; Bjaalie et al., 2005; Koslow and Subramaniam, 2005; Bjaalie, 2008; Tiesinga et al., 2015; Bjerke et al., 2018) to make data discoverable, accessible, interpretable and re-usable, as outlined in the widely endorsed FAIR Guiding Principles (Findability, Accessibility, Interoperability, and Re-usability; Wilkinson et al., 2016). However, these integration efforts face the challenge that neuroscience data span multiple spatial and temporal scales (see, e.g., Amunts et al., 2016), and that results are commonly reported in journal articles as narratives supported with documentation in selected figures and tables that are difficult to compare. A prerequisite for data integration is that the nature and relationships of data parameters are well defined and easily comparable, hence integration efforts will have to incorporate methods for dealing with these differences.

Interpretation of observations collected from the brain depends critically on specific information about anatomical location (see, e.g., Bjaalie, 2002): e.g., from which cortical area, cell layer, or nucleus were measurements or observations obtained? Comparison of results across studies, or replication of experimental findings, necessitates that the specific anatomical position of a measurement, observation, or experimental perturbation, is well-defined. Such anatomical descriptions in experimental reports are of variable quality and are prone to ambiguity, since anatomical terms can be interpreted in a number of ways, and alternative anatomical parcellation schemes often uses different boundary definitions (see, e.g., Van De Werd and Uylings, 2014). Thus, the lack of universally accepted and well-defined descriptions of neuroanatomical location, defining the precise location being studied, is a major challenge when attempting to compare and integrate data from different investigations.

In response to this challenge, open access, three-dimensional (3-D) brain atlases have been developed for murine brains (Hjornevik et al., 2007; Lein et al., 2007; Johnson et al., 2010; Hawrylycz et al., 2011; Veraart et al., 2011; Oh et al., 2014; Papp et al., 2014; Kjonigsen et al., 2015) to serve as spatial frameworks for data sharing and integration (Boline et al., 2008; Amunts et al., 2014; Zaslavsky et al., 2014). Building upon a new generation of 3-D brain atlases, the EU Human Brain Project develops tools and workflows for integrating, sharing and analyzing brain data that have been defined within a common anatomical framework. The project has established workflows for mapping diverse types of murine and human image data to common spatial reference atlas frameworks, building on tools for spatial registration of two-dimensional (2-D) histological image data to a 3-D reference volume (Papp et al., 2016; Puchades et al., 2017), and use of organized collections of metadata describing basic features of data, including descriptions of the anatomical location from which the data originate. These tools and workflows are currently routinely used in the Human Brain Project, but their wider adoption outside the project requires a better understanding of the presently used approaches for describing and documenting neuroanatomical location in experimental studies of murine brains.

To first assess how anatomical location is reported in the neuroscience literature, we evaluated and compared current practices for interpreting and documenting the location of measurements in different disciplines of neuroscience that typically deal with invasive techniques or extraction of tissue. Our observations indicate substantial differences in approach, interpretation and reproducibility of anatomical location between different categories of experimental research, as well as a potential for improvement with relatively simple measures. Based on these observations, we have adjusted and optimized the Human Brain Project tools and workflows to accommodate the type of data and documentation typically used in different domains of experimental research on murine brains. We propose step-wise practical implementations that can improve current practices, and argue that these procedures increase reproducibility and facilitate integration of neuroscience data. We finally discuss costs and benefits of increasingly elaborate approaches.

## Materials and Methods

### Survey of Current Practices for Assignation and Reporting of Anatomical Location

We performed a literature study to explore current practices for reporting anatomical information in different categories of experimental neuroscientific studies in murine brains.

We focused on experimental studies involving invasive procedures or tissue extraction, and classified publications into the following seven methodological categories based on the principal methodology employed: (1) cytoarchitectonic staining techniques, including immunohistochemistry (IHC) and *in situ* hybridization (ISH); (2) axonal tract tracing; (3) transmission electron microscopy (TEM); (4) immunoblotting; (5) *in vitro* electrophysiology with slice preparations and microscopic visualization of recorded cells; (6) *in vivo* electrophysiology, and (7) two-photon and optogenetic imaging. Studies involving tomographic whole-brain imaging and trans-cranial measurements were not included.

Individual search strings were made for each methodological category, and a search was performed in Ovid MEDLINE. Except for the terms related to the specific methods, the criteria used for building the search strings were consistent across searches and contained the following: ((exp mice/or exp rats/) OR (mouse or mice or rat or rats).tw,kf.) AND ((brain or brains or neuroscien^∗^ or neuroanatom^∗^ or neuro anatom^∗^ or neuron or neurons).mp.). Strings related to the specific methodologies of interest (see above) were added to this:

(1) (immunohistochemistry/or immunohistochemistry.tw,kf.) OR ((in situ hybridization).tw,kf.)
(2) ((retrograde or anterograde) adj trac^∗^.tw,kf.) for axonal tract tracing
(3) ((Microscopy, Electron, Transmission/) OR (transmission adj (electronmicroscop^∗^ or electron microscop^∗^)).tw,kf.)
(4) ((western blot^∗^ or immunoblot^∗^).tw,kf.)
(5) ((((invitro or in vitro) adj2 (electrophysiolog^∗^ or electro physiolog^∗^ or cell recording)) or cell recording).tw,kf.)
(6) ((invivo or in vivo) adj2 (electrophysiolog^∗^ or electro physiolog^∗^)).tw,kf
(7) ((optogenetics/or optogenetics or optogenetic^∗^.tw,kf.)) OR ((((twophoton or two photon or two-photon or 2 photon or 2-photon) adj2 (microscop^∗^ or imaging)).tw,kf.))

Filters were then added to limit results to those with journal article format and publication data from 2012 through 15.02.2017. The search returned 9839 entries related to immunohistochemistry and *in situ* hybridization, 547 related to axonal tract tracing, 949 related to electron microscopy, 7004 related to immunoblotting, 95 related to *in vitro* electrophysiology, 213 related to *in vivo* electrophysiology, and 2023 related to optogenetic or two-photon microscopy.

Papers (*n* = 120; 20 for each methodological category) were chosen from the selection of search entries by use of a random number generator and evaluated using the following inclusion criteria: (a) contained murine brain data; (b) presented original data; and (c) were published within the last 5 years. Papers not meeting these criteria were excluded and a new random paper selected.

For each paper in the survey, we evaluated the descriptions of anatomical locations with respect to: (1) any additional information provided beyond the structure name (e.g., by citing an anatomical reference atlas, illustration of the region of interest by use of a schematic drawing or reference atlas plate, or description of the general histological, cytoarchitectonic or electrophysiological features of the region); (2) use of histological sections (without counterstaining); (3) use of (immuno-)histochemical staining to visualize anatomical features; (4) specification of spatial coordinates (e.g., stereotaxic coordinates observed during experimental surgery or by comparison with a reference atlas); (5) documentation using images that show anatomical landmarks suitable for identifying location in addition to features of interest (see below); (6) annotation of anatomical landmarks or boundaries in images from the material; (7) images from multiple (serial) sections through a region of interest; and (8) spatial registration of images to a reference atlas.

Some papers reported results obtained using several methodologies, but for each paper we only assessed the documentation related to the specific methodology for which the paper was selected. Documentation of anatomical location with images was only considered sufficient if images gave a reasonable overview of the regions of interest, allowing the reader to identify the position of the image relative to a reference atlas. We set the minimum standard to be that images should show the region of interest *and* at least one other distinct anatomical landmark, such as a part of the ventricular system, a major white matter tract, or a distinct gray matter structure. Consequently, high-power images showing structural details of a smaller region, e.g., a part of the cerebral cortex with visible layers, were not considered sufficient to allow interpretation of anatomical location in this context.

Most commonly, the region of interest was an *observation site* in which some analysis had been performed (e.g., cell counting, immunoreactivity observations, cell reconstructions). Alternatively, the region of interest may have been a site of an experimental procedure, or *perturbation* (e.g., a lesion, an electrode implantation or a virus injection). In the case of multiple regions of interest of the same type, e.g., cell counting in several regions or multi-site electrode recordings, we assumed the same level of effort had been undertaken to determine the location of each site, and we evaluated the paper according to the best documented region. In the case of multiple regions of interest of different types, we evaluated both types of regions separately. In tract tracing studies, for example, there are sites of perturbation (injection of tracer) and observation (labeled features). For tracing studies we therefore assessed injection sites and terminal fields as individual reports of regions of interest. Thus, while 20 papers from each of the seven methodological categories were surveyed, the total number of papers used was 120, because each tract tracing paper was included in two of the categories (tract tracing injection site and tract tracing terminal fields). References to these 120 papers are given in Supplementary Table 1, which also provides an overview of the observations extracted from 162 different reports of anatomical observations found in these papers.

### Tools Used to Facilitate Documentation of Anatomical Locations

We aimed to demonstrate how new tools and procedures can be applied in order to map and co-visualize data spanning several methodological categories, and to identify key strategies in this process that should influence how data are acquired and documented.

We used the Human Brain Project software tool QuickNII (Puchades et al., 2017) to register single or serial section images to a 3-D reference atlas template by positioning and slicing the atlas in user-defined planes of sectioning. The QuickNII tool is bundled either with the Waxholm Space atlas of the Sprague-Dawley rat brain (version 2, Papp et al., 2014; Kjonigsen et al., 2015)^1^ or the Allen Mouse Common Coordinate Framework (version 3, downloaded June 17, 2016; Oh et al., 2014)^2^. We furthermore used the Human Brain Project tool LocaliZoom for extraction of coordinates from annotated points of interest. The coordinates representing the location of these points in reference atlas space were exported as x, y, z coordinates to MeshView, a Human Brain Project web-viewer tool for visualization of 3-D mesh-data (structural atlas parcellations) together with the point coordinates extracted with LocaliZoom.

### Data Used to Demonstrate Workflows

To demonstrate workflows for spatial integration of different types of data, we used existing or publicly available data sets from the following four methodological categories: (1) *in vivo* electrophysiology; (2) transmission electron microscopy; (3) cytoarchitectonic staining techniques; and (4) *in vitro* electrophysiology with cell reconstruction. These categories represent four of the seven methodologies included in our literature survey, and the workflows used to map these data to anatomical space can easily be extended to the remaining categories.

Electron microscopy data showing parvalbumin positive neurons in the rat medial entorhinal cortex (Berggaard et al., 2018), was generously made available to the present study by Nina Berggaard (Norwegian University of Science and Technology, Norway). *In vivo* electrophysiology recording data from the rat hippocampal region were produced by Debora Ledergerber (Norwegian Institute of Science and Technology, Norway; Puchades et al., 2017). Immunohistological material showing parvalbumin positive neurons across a horizontally cut hemisphere (Boccara et al., 2015) was shared through the Human Brain Project by Menno P. Witter (Norwegian University of Science and Technology, Norway). A 3-D reconstruction of a mouse striatal cholinergic interneuron was performed by Alexander Kozlov, Johanna Frost Nylén and Sten Grillner (Karolinska Institutet, Sweden) and shared via the Human Brain Project^3^. Lastly, a series of sagittal sections from the Allen Institute for Brain Science repository of *in situ* hybridization data (Lein et al., 2007)^4^ was downloaded through their API.

For each data set, section images were spatially registered to a reference atlas template using the QuickNII tool. The first three data sets (*in vivo* electrophysiology, immunohistochemistry, and electron microscopy data) were registered to the Waxholm Space atlas of the rat brain. Section material from the *in vitro* electrophysiology with cell reconstruction and *in situ* hybridization was mapped to the Allen Mouse Common Coordinate Framework of the mouse brain. Following spatial registration to atlas, images with associated atlas information were exported to LocaliZoom for visualization and retrieval of spatial coordinates.

### Ethical Considerations

This study used animal data acquired in accordance with European Union and International legislation regarding use of animal subjects. For data shared by the Human Brain Project, verification of compliance with European legal and regulatory requirements is provided with the data. For other data, statements regarding ethical conduct care are found in the original papers (Lein et al., 2007; Berggaard et al., 2018).

## Results

### Survey of Anatomical Descriptions and Metadata Provided in Original Neuroscientific Reports

We surveyed anatomical descriptions and documentation provided in 120 scientific original reports (published within the last 5 years) involving different types of experimental methods, and evaluated their inclusion of tissue sectioning and histological staining, specification of spatial coordinates, documentation with images (with or without annotations) and the use of spatial registration to anatomical reference atlases. We found systematic variations across methodological categories regarding the degree to which anatomical locations were described and documented (summarized in Table 1). Below, we first summarize our findings of anatomical documentation per methodological category, and secondly compare the use of anatomical descriptions and different types of documentation across the methodological categories.

**Table 1:** Overview of anatomical metadata elements provided in the publications investigated.

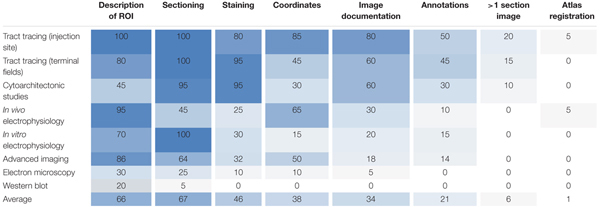

*Percentage of papers (*n* = 120), sorted by methodological category (rows), using different approaches (columns) to document anatomical location selected articles. Employed practices to identify and document anatomical location vary across the categories of investigations. Most studies describe location semantically, while the use of image documentation and spatial registration of images to reference atlases is limited.*

Tract-tracing studies generally provide more anatomical documentation than studies using other methods (Table 1). In 85% of the papers investigated, the location of tracer injection sites was given by the perioperatively recorded stereotaxic coordinates used when placing the tracer injections, and 35% of these further documented injection sites with section coordinates upon verification of position. Injection sites were often documented with images showing anatomical location of the injection (80%), and in 60% of cases the regions of interest containing neuronal labeling were also documented with images (Table 1). Contrary to our expectation, we found that in most of the investigated papers reporting on tract tracing experiments (17 of 20 papers, 85%), the anatomical location of tracer injection sites was more thoroughly described and documented than the location of transported neuronal labeling in one or more remote brain regions (see Table 1 for details).

While all reports from cytoarchitectonic investigations used histological techniques and almost all (18 of 20, 90%) presented images of microscopic observations, only 60% provided images with visible anatomical landmarks (Table 1), and very few (10%) included images showing their region(s) of interest across multiple sections. In several of the papers investigated we found it difficult to interpret and reproduce the investigated regions in a reference atlas.

Our results also show that *in vivo* electrophysiological experiments typically provide better documentation of anatomical location than most other study types, mainly as the location of the recording electrodes (in 65% of cases, Table 1) is usually defined by perioperatively recorded stereotaxic coordinates. In 38% of these papers, implantation sites were further documented by providing histologically verified section coordinates. Of the publications reporting on *in vitro* electrophysiological studies with microscopic visualization of recorded cells, 30% used histological staining to reveal anatomical landmarks, but only 20% included overview images documenting anatomical boundaries or landmarks (Table 1).

Studies using advanced *in vivo* optogenetic or two-photon microscopic imaging techniques often (in 85% of cases, Table 1) contained some form of description of the region of interest, and coordinates were provided in approximately half of the papers. Somewhat surprisingly, we found that the microscopic images of the analyzed material rarely (in 18% of the papers investigated, Table 1) included anatomical landmarks suitable for documenting anatomical locations.

Lastly, we found that documentation of anatomical location was, to a small degree, provided in reports of electron microscopy and immunoblot studies. 20–30% of such studies contained anatomical descriptions of regions of interest, while use of additional documentation was minimal or absent (Table 1). These types of studies were the least likely to include sufficient anatomical information of all the assessed methods.

Our results thus show that of all 120 papers surveyed only 66% included some form of anatomical descriptions of regions of interest, beyond mention of the region name (Table 1). In the remaining 34% of papers, we found no descriptions of the region of interest apart from the name of the region. A further breakdown of the 66% papers providing anatomical descriptions is summarized in Table 2. This breakdown showed that 29% of the papers providing a description of a region of interest did so by using a reference to a specific anatomical atlas. Notably, we also found that anatomical reference atlases were most frequently cited in reports of tract tracing injection sites (60%, Table 2), while none of the immunoblot reports providing anatomical descriptions related these to a reference atlas. Interestingly, among the anatomical descriptions provided in reports of neural labeling observed in tract-tracing studies, or advanced imaging studies, only 44 or 11%, respectively, included reference to a specific anatomical atlas. We further found that among the 66% of papers including anatomical descriptions, 76% included illustrations or line drawing of anatomical features, 42% indicated measurements of distances to specific anatomical landmarks, and 13% related their descriptions to observed microscopic or electrophysiological features (Table 2).

**Table 2:** Overview of the types of descriptions and coordinate based information provided the publications investigated.

	Tract tracing injection site	Tract tracing terminal fields	Cytoarchitectonic studies	*In vivo* electrophysiology	*In vitro* electrophysiology	Advanced imaging	Electron microscopy	Western blot	Average
**Descriptions**	**100**	**80**	**45**	**95**	**70**	**86**	**30**	**20**	**66**
Based on distance to landmark	90	13	22	84	0	68	33	25	42
Based on reference atlas	60	44	22	53	21	11	17	0	29
Based on illustration	55	100	89	58	71	89	67	75	76
Based on cellular features	20	19	0	26	36	5	0	0	13
**Coordinates**	**85**	**45**	**30**	**65**	**15**	**50**	**10**	**0**	**38**
Point coordinates	100	0	0	100	0	91	50	0	43
Section coordinates	35	45	100	38	100	27	50	0	49


*Percentage of papers (*n* = 120) from different methodological categories (columns) providing different types of documentation (rows) as descriptions or point coordinates.*

Although stereotaxic atlases are widely used and stereotaxic coordinates provide precise indications of location, we found that coordinate-based information was presented in only 38% of the papers surveyed (Table 1). Further breakdown showed that of the 38% of papers that included spatial coordinates, 49% provided positions of sections or slices reported as distances from skull landmarks or the midline (Table 2), while 43% specified points of interest as x, y, z coordinates (Table 2) targeted perioperatively and/or identified by *post hoc* analyses. Of the publications reporting tract tracing or *in vivo* electrophysiological experiments, 65% included spatial coordinates, while none of the publications reporting immunoblotting results contained such information.

While most studies included high-power image documentation of observed features, only 34% included images showing anatomical landmarks and/or boundaries suitable for interpretation of anatomical locations, while 21% of studies provided images with anatomical annotations superimposed (Table 1). Most of the images showing anatomical landmarks were restricted to one brain region of interest; in fact, only ∼9% of all studies provided overview images showing a whole, half or smaller part of a brain section. None of the papers examined included images from macroscopic dissection. Only 6% of the papers used more than a single section image to document the same region of interest (Table 1).

Only two of the surveyed papers (1%) used spatial registration tools to map the position of their experimental images to anatomical reference atlases (Table 1). We thus found systematic differences in the documentation of anatomical regions of interest provided in original research papers that varied across methodological subfields of neuroscience. Our findings indicate that most studies lack elementary descriptions and documentation of anatomical location that in principle should be straightforward to include in scientific reports, regardless of the type of methodology used.

### Minimum Requirements for Documentation of Locations in Experimental Murine Brain Research

Based on the above findings, we considered how anatomical descriptions from different methodological traditions could be improved to achieve more consistent and reproducible descriptions of anatomical locations. A key principle underlying empirical scientific research is that original publications should contain sufficient descriptions of materials and methods used to allow peers to reproduce experimental results. Extrapolating from this, an obvious minimum requirement for the reporting of anatomical location is that anatomical regions of interest should be specifically and unambiguously reported, with sufficient documentation to allow interpretation and replication of described anatomical positions. However, our survey of the current literature above revealed that the location of data is often poorly described and documented, making reported anatomical positions hard to replicate. Combining the findings summarized in Table 1 and accumulated experiences with interpretation and validation of anatomical locations in a wide range of materials measurements collected in context of the Human Brain Project (see e.g., Bjerke et al., 2018), we identified some key documentation elements that we found to be of particular importance for our ability to unequivocally specify anatomical locations for different data sets. We also formulate a set of method-independent recommendations for a minimum documentation practice that could alleviate the ambiguity observed in many research papers (see above), and facilitate interpretation of anatomical positions and comparison of research findings. Thus, to achieve more unambiguous and reproducible descriptions of anatomical locations in neuroscientific reports we propose that adherence to at least one and preferably several of the following recommendations should be set as a minimum requirement:

(1) Employ and refer to a specific anatomical parcellation scheme
(2) Provide precise semantic descriptions relating observations to anatomical landmarks or features
(3) Define points or regions of interest using an anatomical illustration or diagram
(4) Provide annotated images showing distinct anatomical landmarks
(5) Report spatial coordinates

Below, we specify and exemplify these recommendations in further detail.

#### Referring to a Defined Parcellation Scheme

**FIGURE 1:**
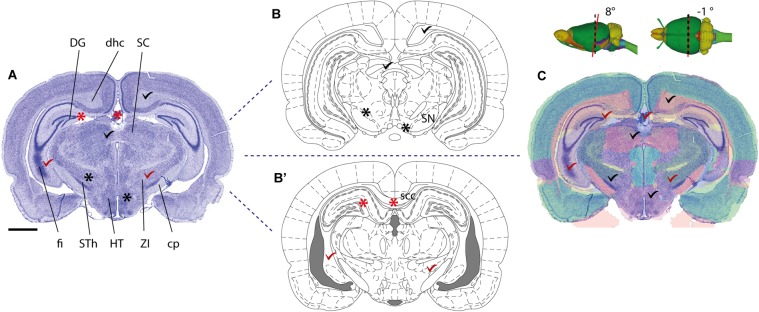
Mapping a coronal rat brain section with a slight tilt to a reference atlas**. (A)** Image of a thionine stained coronal rat brain section, cut with a slight deviation from the standard plane of orientation. A slight left-right asymmetry is visible in the fimbria of the hippocampus (fi), and when comparing the section with a standard reference atlas, reference plates from different anteroposterior levels match the dorsal and ventral parts of the section, respectively. **(B,B’)** Atlas diagrams 78 **(B)** and 68 **(B’)**, reproduced from Paxinos and Watson (2005) with permission, separated by ∼1.5 mm. In both diagrams, anatomical structures that are consistent (check marks) or inconsistent (asterisks) with corresponding regions in the histological section are seen. Regions located dorsally in **(A)**, such as the dentate gyrus (DG), dorsal hippocampal commissure (dhc) and the superior colliculus (SC) correspond with plate 78 **(B)**, but not plate 68 **(B’)**, while regions located ventrally in **(A)**, such as the fimbria (fi), subthalamic nucleus (STh), hypothalamic region (HT), zona incerta (ZI), and cerebral peduncle (cp) correspond with plate 68 **(B’)**, but not with plate 78 **(B)**. **(C)** A custom generated transparent atlas overlay from the 3-D Waxholm Space atlas of the rat brain (v2, Papp et al., 2014; Kjonigsen et al., 2015) superimposed onto the thionine section shows better overall anatomical correspondence of structures in both dorsal and ventral parts of the section. The location and tilt of the custom atlas plate is indicated by red lines in the inset 3-D figures. Scale bar, 2 mm.

Several parcellation schemes exist for the whole mouse and rat brain, including the widely used 2-D stereotaxic reference atlases (Swanson, 2004; Paxinos and Franklin, 2012; Paxinos and Watson, 2013), and 3-D reference atlas templates (Johnson et al., 2010; Hawrylycz et al., 2011; Papp et al., 2014). More detailed parcellation schemes have also been defined for parts of the brain, such as the hippocampus (Kjonigsen et al., 2011; Witter, 2012; Boccara et al., 2015). Anatomical parcellation schemes should preferably include graphical representations of the boundaries defining anatomical structures specified in a nomenclature list. Use of standardized schemes that are widely used in the community will facilitate comparison of anatomical locations. To be unambiguous, a description based on a parcellation scheme should include (1) the name of the region of interest exactly *as it appears* in the reference atlas, and (2) appropriate citation of the reference atlas (and version) employed. Reference to 3-D atlas templates, e.g., the Allen Mouse Common Coordinate Framework (Lein et al., 2007; Oh et al., 2014) or Waxholm Space (Hawrylycz et al., 2011; Papp et al., 2014), that can be sliced in any orientation provides superior anatomical precision for both volumetric data and 2-D sectioned data (Figure 1). For observations or measurements that are sampled from an entire brain region, for example describing populations of labeled cells distributed across a given brain region, reference to the region name will usually be unambiguous. However, if observations or measurements only pertain to a small subset of a region of interest, e.g., for a single cell reconstruction or a tissue sample processed for electron microscopy, information about parcellation scheme should be supplemented with one of the other recommendations listed above to more clearly specify location within the region of interest, e.g., using an image or anatomical illustration.

#### Semantically Describing Spatial Relation to Distinct Anatomical Landmarks or Architectonic Features

In some cases, a region of interest might be described by defining its relation to structural or cellular landmarks. This is a particularly relevant form of description when regions are defined differently or with more detail than in standard atlas frameworks. See, for example, Insausti et al. (1997, pp. 151–155), where subregional boundaries of the entorhinal cortex are described both in terms of cytoarchitectonic features, and in relation to anatomical landmarks. It should be emphasized that while such description can be elaborate and detailed, they can also be challenging to interpret without expert knowledge. Anatomical illustrations or annotated images can facilitate easier interpretation for the reader.

#### Indicating the Location in an Anatomical Illustration

Anatomical locations can be graphically defined using reference atlas diagrams, schematic summary drawings or other figures. Indication of sampling position within such illustrations (see, e.g., Akhter et al., 2014, their Figure 2) can serve as supplements to semantic descriptions, or alternative to spatial coordinates.

**FIGURE 2:**
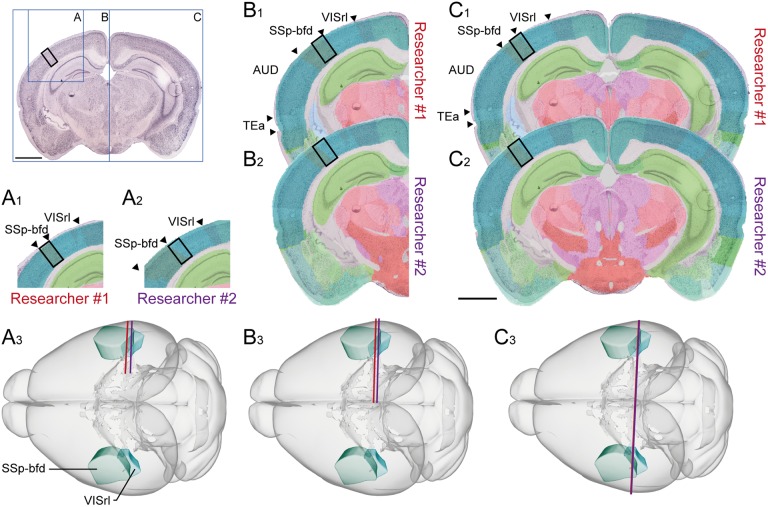
Documenting anatomical location at varying levels of coverage. Illustration showing the influence of image coverage on the spatial accuracy of registration to a reference atlas. Upper left inset: image of a coronal, thionine stained mouse brain section, the black rectangle indicates an arbitrary region of interest in the cerebral cortex, blue rectangles indicate the size of the image used in examples **(A–C)**. **(A–C)** Images with increasing anatomical coverage were shown to two experienced researchers, who independently registered the images to the Allen Mouse Common Coordinate Framework using the QuickNII tool. **(A)** With only a small part of the section image available, the two researchers interpreted the location of the image differently, as the primary somatosensory cortex, barrel field (SSP-bfd, **A1**) by Researcher #1, and as the rostrolateral visual area (VISrl, **A2**) by the Researcher #2. (**A3**) Shows the positions of the section image assigned by Researcher #1 (red line) and Researcher #2 (purple line) in atlas space. **(B)** With an image showing half of the section, the researchers’ interpretations of the position become more similar **(B1–B3)**, and when the entire section image was available, both researchers interpreted the region of interest to be located in SSp-bfd **(C1–C3)**. This illustrates that access to images covering more anatomical landmarks is important to provide reproducible information about anatomical positions. Scale bars: 2 mm.

#### Providing (Annotated) Images

**FIGURE 3:**
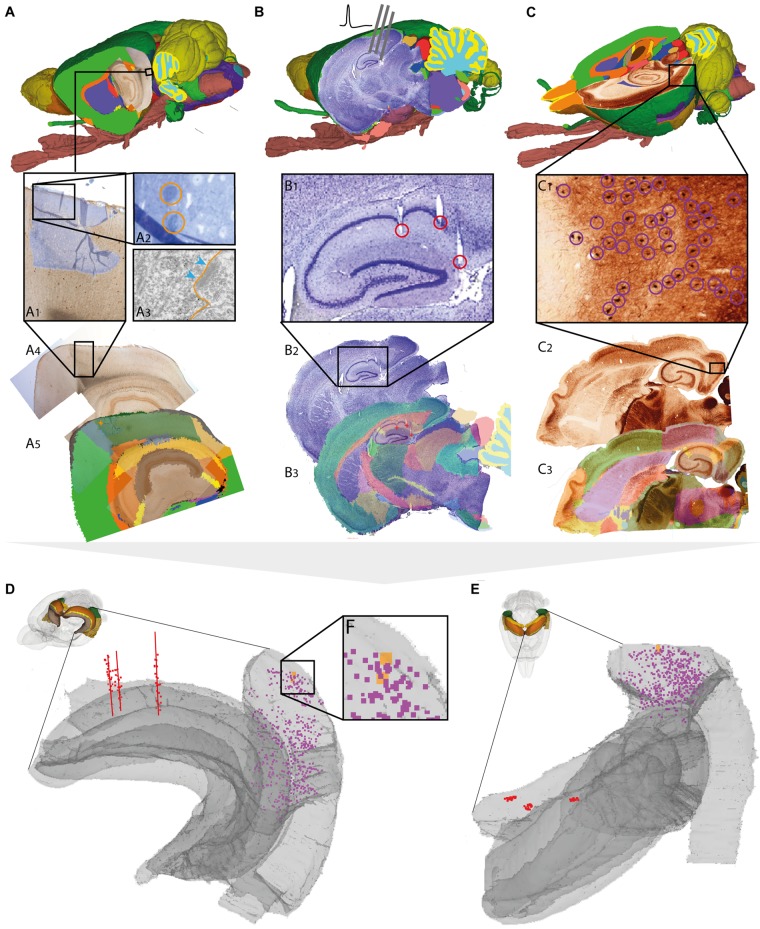
Assigning anatomical location and integrating data using 3-D atlas and new workflows: Examples showing rat brain data. **(A–C)** Data from experimental studies using **(A)** transmission electron microscopy (Berggaard et al., 2018), **(B)** *in vivo* electrophysiology (Puchades et al., 2017), and **(C)** histochemical visualization of chemoarchitecture (Boccara et al., 2015), are mapped to the Waxholm Space atlas of the rat brain (v2, Papp et al., 2014; Kjonigsen et al., 2015). **(A1–A5)** Show the stepwise procedure used to define the location of an electron microscopy (EM) image in atlas space, by mapping an image of semithin, toluidine blue-stained section onto a low-power image showing a larger part of the brain **(A1)**. In this way the location of the parvalbumin stained cell (encircled in **A2**) shown in the EM image **(A3)** is determined in different images. By mapping the overview image **(A4)** in atlas space **(A5)**, the location of the paravalbumin positive cells shown in **(A3)** can be defined by 3-D atlas coordinates (orange crosses in **A5**). **(B1–B3)** Show how tracks of recording electrodes (encircled in **B1**), visible in a thionine stained section cut obliquely halfway between the coronal and sagittal plane **(B2)**, are mapped in atlas space by registration to the reference atlas (red crosses in **B3**). **(C1–C3)** show how positions of parvalbumin positive cells (encircled in **C1**), visualized by immunolabelling of horizontal rat brain sections **(C2)** can be determined by spatially registering section images to a reference atlas **(C3)**. **(D,E)** 3-D co-visualization of point coordinates extracted from the three data sets (**A**, orange dots; **B**, red dots, **C**, purple dots) together with gray surfaces of the right hippocampal and entorhinal regions, shown from an anterolateral **(D)** and dorsal **(E)** view. **(F)** Magnified view of data points representing the location of the cells shown in example **(A)**.

Images of experimental material may depict sections or macroscopic dissections. Low-resolution images are generally as useful for the purpose of visualizing location as high-resolution ones, even images obtained with a standard cell phone camera. Section images should show structural landmarks outside the region of interest, if possible more than one. Suggestions for anatomical landmarks that can be consistently identified in volumetric material of the rodent brain are provided in Sergejeva et al. (2015). For sectioned material, the size and shape of prominent gray and white matter regions (e.g., the hippocampus, caudoputamen, pontine nucleus, anterior commissure, and corpus callosum) are also highly useful in order to interpret location in the brain. Ideally, images should cover entire sections. A simple evaluation of the influence of the coverage of a section image on spatial registration accuracy (Figure 2) confirms that the more information an image contains, the more likely are two independent and equally experienced researchers to interpret the anatomical position of a region of interest consistently. For procedures not involving histological processing and tissue sections, macroscopic images of the whole brain or tissue sample(s) before and after dissection of tissue samples can improve the interpretation of the anatomical location of the investigated sample considerably (Figure 3A). Annotations defining regional boundaries and specifying locations sampled or measured increase precision considerably (see, e.g., Dobi et al., 2013; their Figure 9).

#### Using Spatial Coordinates

Spatial coordinates defined in relation to unique skull features or anatomical landmarks effectively communicate exact positions within the brain, independent of parcellation schemes. Descriptions based on spatial coordinates must specify the reference space used (e.g., reference atlas or local coordinate system). Coordinates may indicate the level or distance of a section or slice from an anatomical landmark (e.g., bregma or the midline of the brain) or specific points defined by x, y, z coordinates. The method used to define coordinates should be specified and additional validation steps, such as e.g., histological confirmation of perioperative stereotaxic measurements supplemented with documentation using image(s) or illustrations, can improve precision.

### Combining Different Types of Data in Reference Atlas Space

A considerable challenge for efforts toward integration of different types of neuroscience data is the heterogeneity in the spatial scale and modality of data. In context of the ambition of the Human Brain Project to make heterogeneous brain data accessible for integrative analyses and computational modeling (Bjerke et al., 2018), we have explored ways to assign location to disparate categories of murine neuroscience data. Using the minimum requirements for documentation of anatomical location proposed above as a starting point, and having the ambition to optimize anatomical descriptions of different types of neuroscience data, we established workflows to relate data sets acquired by *in vivo* electrophysiology, immunohistochemistry, *in situ* hybridization, transmission electron microscopy and *in vitro* electrophysiology with cell reconstruction to a common spatial atlas framework. The core workflow, used to spatially combine features of interest from different types of data sets, involves three steps. We first link the data to the same anatomical reference framework, secondly extract spatial coordinates representing features of interest from each of the data sets and thirdly co-visualize the extracted features in a 3-D atlas viewer as a starting point for various analytic approaches. The workflow is implemented using a suite of digital atlas and viewer tools developed in the Human Brain Project. The QuickNII tool is developed for registering 2-D (serial) images to a reference atlas by mapping a spatially corresponding, customized atlas image onto images (Puchades et al., 2017). The LocaliZoom viewer tool provides an overlay of custom made reference atlas maps and allows extraction of spatial coordinates representing features of interest. The 3-D viewer tool MeshView was used to co-visualize the color coded coordinates from different data sets together with selected elements from the 3-D reference atlas. With this core workflow as a basis, we identified specific strategies for determining anatomical location and extracting spatial coordinates for features of interest from each methodological category. The step-wise implementation of these workflows are exemplified below for different types of data, illustrating how descriptions of location can be improved and used for data integration purposes with relatively simple steps.

For *electron microscopy* data, spatial coordinates were obtained for two parvalbumin positive cells from the medial entorhinal cortex, imaged under a transmission electron microscope (Figure 3A3). The ultrathin sections used for electron microscopy were sectioned from a small tissue sample dissected from a sagittal vibratome rat brain section from the temporal cortex, stained for parvalbumin by immunohistochemistry. Prior to ultrathin sectioning, semithin sections were obtained and counterstained using toluidine blue (Berggaard et al., 2018) for orientation and identification of immunopositive cells. To determine the location of the cells viewed by electron microscopy in a 3-D reference atlas, three main steps were followed (Figures 3A1–3A5). First, an image of the entire sagittal brain section taken prior to removal of the tissue sample was mapped to the reference atlas using QuickNII (Figure 3A5). Secondly, a transparent image of the semithin, toluidine blue-stained section was manually registered to the larger image of the sagittal section, by aligning specific features visible in both images, including blood vessels, labeled cells, outer surface and boundary between gray and white matter (Figure 3A1). Finally, the location of the parvalbumin positive cells was identified both in the semithin and ultrathin sections, and coordinates were extracted from the vibratome section (Figure 3A5), thus allowing identification of cells across all spatial scales. The above procedure can in principle be applied to any method involving small tissue samples, such as immunoblotting and related methods.

For *electrophysiological recording* data, spatial coordinates were extracted from the bottom of individual electrode track throughout a series of sections cut in a non-standard plane and stained to reveal cytoarchitecture (Figure 3B; Puchades et al., 2017). While the location of electrophysiological recordings is usually reported by use of perioperatively determined stereotaxic coordinates (Table 1; see above), a key step to improve precision is to determine the location of electrode tracks in histological sections. In our example, a non-standard oblique section plane was used to identify electrode tracks, which is very difficult to compare with a traditional 2-D atlas framework. Using the QuickNII tool, the section image could nevertheless be mapped to atlas space, thus allowing the location of electrode tracks to be annotated and visualized (Figures 3B1–3B3; Puchades et al., 2017; see also similar example shown in Bjerke et al., 2018).

**FIGURE 4:**
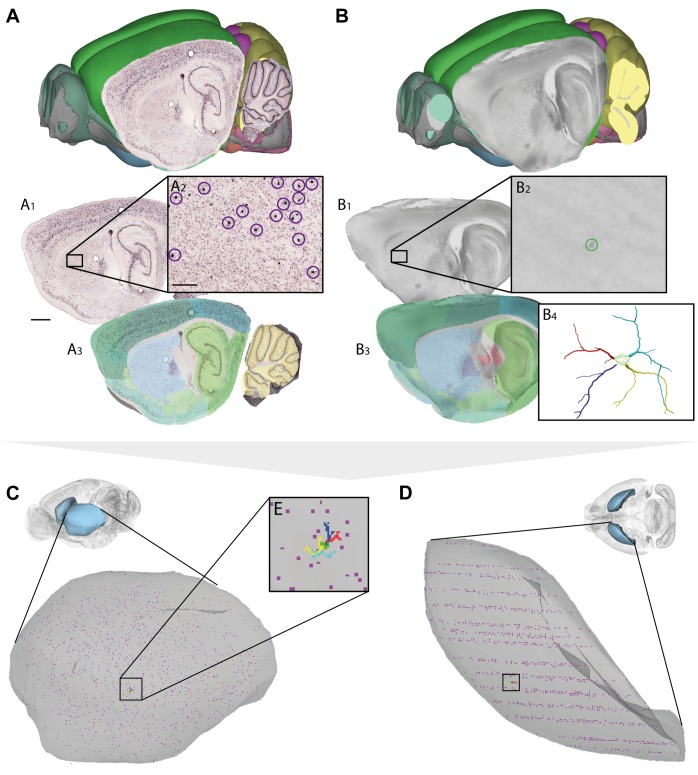
Assigning anatomical location and integrating data using 3-D atlas and new workflows: Examples showing mouse brain data. **(A,B)** Data from experiments using **(A)** *in situ* hybridization (Lein et al., 2007) and **(B)** *in vitro* electrophysiology with single-cell reconstruction (Kozlov et al., unpublished data) mapped to the Allen Mouse Common Coordinate Framework. **(A1–A3,B1–B4)** show the stepwise procedures used to map overview images covering sagittal sections **(A1,B1)**, showing labeled cells of interest (encircled in **A2,B2**), to reference atlas space **(A3,B3)**. **(B4)** shows the 3-D reconstruction of the neuron shown in **(B2)**. **(C,D)** 3-D co-visualization of point coordinates extracted from the two data sets (**A**, purple dots; **B**, red, green, blue, and yellow dots) together with the surface of the caudoputamen, shown in view from anterolateral **(C)** and dorsal **(D)**. **(E)** Magnified view of data points representing the location of the 3-D cell reconstruction shown in example **(B)**. Scale bars: 1 mm **(A1)**, 200 μm **(A2)**.

For *histological material* used in microscopic studies of brain architecture, the strategy for extracting coordinates for labeled features of interest is straightforward compared to the examples above. In our example (Figure 3C), we used images of serial histological sections immunostained for parvalbumin (Boccara et al., 2015). After mapping the serial section images to the reference atlas, we recorded point coordinates representing immunopositive cells located within the medial entorhinal cortex in sections sampled at 200 μm intervals through the entire left entorhinal cortex (Figure 3C1). A similar example is shown in Figure 4A, using section images (downloaded from the Allen Institute for Brain Science, Lein et al., 2007)^5^ showing parvalbumin positive cells visualized by *in situ* hybridization. The spatial registration of these images to the Allen Mouse Common Coordinate Framework was adjusted using the QuickNII tool, and point coordinates representing parvalbumin positive cells in the left caudoputamen were extracted from all sections (Figure 4A2).

For *neuron reconstructions*, a slightly different approach was used. The data included coordinate lists created by 3-D reconstruction of neurons (intracellularly filled with neurobiotin) using the Neurolucida software tool (MBF Bioscience, Williston, VT, United States), together with low-power images of sagittal sections images in which the labeled somata were visible. The sagittal section images were registered to the mouse brain atlas using QuickNII, following which the atlas coordinates corresponding to the center of the neuronal soma (seen in the histological section, cf. Figure 4B2) were extracted using LocaliZoom. Having determined the center point of the soma and the position and orientation of the histological section image in atlas space, we spatially translated the local (Neurolucida) coordinates representing the complete neuronal arbors of the 3-D reconstructed cell to atlas coordinates.

Thus, by mapping very different types of data to a common anatomical reference atlas, it became possible to extract point coordinates for key data features and co-visualize these in atlas space (Figures 3D,E, 4C–E).

### Improving Location Metadata Using New Tools and Workflows for Anatomical Localization

Based on the strategies and documentation elements used above to connect different types of data to a common anatomical framework, and extending on the minimum practice recommendations proposed above, we suggest the following additional method-independent documentation steps to improve precision and facilitate data integration: (1) document features of interest in relation to cellular or regional characteristics; (2) register images to a 3-D reference atlas framework; and, if possible (3) acquire multiple serial histological (or tomographical) images covering several anatomical landmarks.

#### Document Features of Interest in Relation to Cellular or Regional Characteristics

The anatomical boundaries of brain regions are usually defined by characteristic structural or functional features. These can be visualized by (immuno-)histological staining, such as the thionine or parvalbumin staining shown in Figure 3, or other cellular properties such as autoradiographic visualization of receptors (Schubert et al., 2016) or enzyme based visualization of chemical properties (e.g., patches of cytochrome oxidase positive cell groups in the sensory whisker barrel cortex; Land and Simons, 1985). Additional approaches used to pinpoint anatomical location include, e.g., visualization of specific well-known cellular architectures or connectivity, electrophysiological measures of sensory receptive fields (Chapin and Lin, 1984) or motor-related activity (Neafsey et al., 1986), or any combination of the above. Use of such measurements can allow more fine-grained and precise anatomical descriptions of location.

#### Spatial Registration of Images to Reference Atlas

As shown in the examples provided in Figures 3, 4, spatial registration of brain images to an anatomical atlas provides specific evidence of location in a standard anatomical reference space. If images have been acquired with orientations matching the standard coronal, sagittal and horizontal planes used in reference atlases, such registration can simply be done by mapping 2-D diagrams from any standard reference atlases on section images. However, to correct for deviations in the angle of orientations commonly seen in histological sections, and to more directly relate positions in the experimental images to a spatial 3-D reference framework, we recommend mapping images to a 3-D atlas (Lein et al., 2007; Hawrylycz et al., 2011; Oh et al., 2014; Papp et al., 2014). Depending on the properties of the experimental material used, several software tools are available for such purposes (Majka and Wójcik, 2016; Puchades et al., 2017; Figures 3, 4), allowing more accurate determination of the anatomical position and section angle in experimental material (Figure 1B).

#### Use of Serial Sections

Interpretation of anatomical location, particularly for the purpose of spatial registration, can be improved with use of serial section images that display multiple anatomical landmarks. Inclusion of more sections is particularly useful when determining deviations of section angles from the standard plane. The precision of such a registration can therefore be improved by including more section images than the ones used for analysis.

Regardless of the atlas used and the methods for relating data to it, the anatomical information as extracted from the atlas (region names, coordinates of points or sections) for the entire analyzed region(s) should be clearly communicated in publications and collections of metadata.

## Discussion

We have here reviewed anatomical location metadata provided in recent neuroscience publications, and found considerable differences across subfields of neuroscience. We have proposed a set of method-independent, easily adopted practices (minimum requirements) that can significantly improve reproducibility of neuroanatomical locations reported in publications. Furthermore, we have shown that re-usability and integration of data can be improved with additional steps using new software tools and workflows developed through the Human Brain Project, and that these procedures are applicable to data obtained by a range of methods.

Factors contributing to inconsistency and ambiguity in location metadata included (1) variable use of reference atlases, (2) lack of specification regarding nomenclatures, terms, and definitions used, (3) limited use of coordinate-based information, and (4) use of highly magnified image material without sufficient annotation as the only graphical display of data location. The amount of location metadata found in publications depended on the methodology with which the data had been obtained, likely pointing to different approaches and traditions having evolved as common practice within subfields of neuroscience.

The minimum requirements presented here are intended to be flexible and easily applicable to any neuroscientific method. They essentially state that descriptions of locations should be complete, and precisely define the relationship of sites of interest to anatomical landmarks, by use of semantics, coordinates or graphical representations, and preferably a combination of these. Appropriate reference to a specific nomenclature and citation of the reference atlases consulted is an obvious requirement, which is easy to implement regardless of the method used, but as our results show, often overlooked. We claim that adherence to the minimum recommendations requires little additional effort by researchers, and can substantially improve the precision of anatomical descriptions and data interpretation in neuroscience publications. Our examples specify how this can be implemented for different types of data.

However, comparison of descriptions based on text, reference atlases and image material remains dependent on substantial human interpretation. The second part of our work therefore demonstrate that data obtained by several methodologies, spanning spatial and temporal scales, may be thoroughly and accurately located in space using novel tools and workflows, and that the output of these procedures can be used to co-visualize data.

The workflows tested here for mapping data to atlas space can be implemented for any neuroscience method, provided that image material showing features of interest in relation to anatomical landmarks is available. For methods where such features are readily seen in histological section images, the procedures are quite easily applicable, as seen in our examples using immunohistochemical and *in situ* hybridization material, as well as *in vivo* electrophysiological recordings. In the case of electron microscopy data, the spatial correlation of features seen at the microscopic and ultrastructural levels is essential in order to map specific objects imaged at the electron microscopic level to a reference atlas. This was achieved here using low-power overview images acquired during tissue processing and images of semithin sections stained to show cytoarchitecture. Some steps could have been improved, e.g., by imaging the whole brain section before and after sectioning, and by keeping track of the location within the ultrathin section from which the electron microscopy images were obtained. An alternative approach would be to extract coordinates representing the perimeters of the data set, e.g., the corners of a block of tissue dissected from a vibratome section and prepared for electron microscopic imaging. Whether highly specific information about the position of individual cellular elements is desirable and attainable for a data set will depend on the research question and the methods of tissue preparation. Nevertheless, our example shows that even minor additions to common protocols (e.g., acquiring images of sections from which electron microscopic samples have been dissected) can give major improvements of precision of location metadata. For neuronal reconstruction data, we show that spatial coordinates recorded with a 3-D reconstruction software can be translated to atlas coordinates by using reference points visible in macroscopic section images. For mapping of more complex neuronal arbors in atlas space, annotation of at least four (and preferably more) reference points representing key landmarks in the neuronal reconstruction will increase precision. Again, access to low-power overview images documenting soma locatio relative to visible landmarks was critical for translating the location of reconstructed neurons to atlas space.

We lastly summarized the workflows developed through these examples as a set of improved practices, aimed to facilitate efforts to compare and integrate neuroscience data. Mapping data to a common anatomical framework is an effective means to allow comparison and facilitate integration of disparate data types, a key goal within the Human Brain Project (Bjerke et al., 2018). Adherence to the improved practice recommendations proposed here ensures that heterogeneous data can be organized and shared in databases, with location metadata suitable for conducting queries based on location, either by using semantic strings and anatomical ontologies or by use of more fine-grained 3-D spatial queries for coordinate locations in atlas space.

Additional documentation and more extensive interpretation of anatomical locations, as exemplified above, requires additional efforts including production of additional material and documentation, as well as analytical efforts. Depending on the size of the data set, type and quality of images and the features to be extracted, the process of registering data, extracting and visualizing coordinates requires from a couple of days to a week. The workflows used to extract spatial coordinates for different data features in our examples, were based on manual annotations performed with the tool LocaliZoom. The advantage of this approach is that atlas coordinates are directly exported, but it can be tedious to apply to larger data sets. New tools and workflows are currently being developed in the Human Brain Project that will allow (semi-)automated extraction of labeled features from serial images (Kreshuk et al., 2014; Papp et al., 2016; Yates et al., 2017).

We argue that costs of such additional efforts are outweighed by improved precision of anatomical location metadata, and the added value gained by making data easier to compare across studies. Today, finding and comparing data in the literature based on a region of interest is a time-consuming task that often reveals inconsistencies in results. Indeed, flexible use of definitions has been related to poor reproducibility in science (Ioannidis, 2005). Concepts of brain regions are examples of such fluid definitions (Van De Werd and Uylings, 2014), and inaccurate reporting of location is likely to amplify the challenge caused by these changes. However, the coordinate systems that embed concepts of brain regions are static. Mapping current data to such coordinate frameworks will make data more robust in the face of evolving concepts of brain regions and is thus necessary to ensure long-term relevance of findings. Furthermore, following the improved practice recommendations outlined here can facilitate data integration and re-use of data, as the output of spatial registration procedures is structured metadata about anatomical locations that can accompany data to be shared. We therefore consider the benefits of performing these methods to outweigh the costs in the long term. New practices for data sharing in neuroscience (Ferguson et al., 2014; Leitner et al., 2016; Ascoli et al., 2017) will likely lead to augmented focus on high-quality metadata as a tool for increasing the value and impact of data, and thus also establish more prominent short-term incentives for mapping data to reference atlas space.

## Data and Software Availability Statement

The data sets generated for the literature survey of this study are available from the corresponding author upon request. The data sets used for testing procedures for spatial registration were obtained from public sources (Allen Institute for Brain Science repository, the Human Brain Project), or from generous colleagues as specified in the methods section. Requests to access these data sets can be directed to t.b.leergaard@medisin.uio.no. Software tools are available from the Human Brain Project (www.humanbrainproject.eu), requests for access to these tools can be directed to j.g.bjaalie@medisin.uio.no.

## Author Contributions

IB contributed to conceiving the study, performed analyses, contributed to development of workflows, and co-authored the manuscript. MØ and KA contributed to conceiving the study, performed analyses, and contributed to development of workflows. CB, HK, SY, and MP contributed to the analyses and development of workflows. JB supervised development of tools, infrastructure, and workflows and contributed to writing the paper. TL conceived and supervised the study, and co-authored the manuscript. All the authors reviewed and approved the manuscript.

## Conflict of Interest Statement

The authors declare that the research was conducted in the absence of any commercial or financial relationships that could be construed as a potential conflict of interest.

## Footnotes

1 https://www.nitrc.org/projects/whs-sd-atlas

2 http://download.alleninstitute.org/informatics-archive/current-release/mouse_ccf/annotation/ccf_2015/

3 https://www.humanbrainproject.eu/en/explore-the-brain/search/

4 http://mouse.brain-map.org/experiment/show/75457579

5 http://mouse.brain-map.org/experiment/show/75457579
